# Predicting Dominant Genotypes in Norovirus Seasons in Japan

**DOI:** 10.3390/life13081634

**Published:** 2023-07-27

**Authors:** Yoshiyuki Suzuki

**Affiliations:** Graduate School of Science, Nagoya City University, 1 Yamanohata, Nagoya-shi, Aichi-ken 467-8501, Japan; yossuzuk@nsc.nagoya-cu.ac.jp; Tel.: +81-52-872-5821

**Keywords:** genotype, norovirus, P-type, prediction, proportion, recombination

## Abstract

Human noroviruses are an etiological agent of acute gastroenteritis. Since multiple genotypes co-circulate every season changing their proportions, it may be desirable to develop multivalent vaccines by formulating genotype composition of seed strains to match that of dominant strains. Here, performances of the models for predicting dominant genotypes, defined as the two most prevalent genotypes, were evaluated using observed genotype frequencies in Japan and genomic sequences for GI and GII strains. In the null model, genotype proportions in the target season were predicted to be the same as those in the immediately preceding season. In the fitness model, genotype proportions were predicted taking into account the acquisition of novel P-types through recombination and genotype-specific proliferation efficiency, as well as herd immunity to VP1 assuming the duration (*d*) of 0–10 years. The null model performed better in GII than in GI, apparently because dominant genotypes were more stable in the former than in the latter. Performance of the fitness model was similar to that of the null model irrespective of the assumed value of *d*. However, performance was improved when dominant genotypes were predicted as the union of those predicted with *d* = 0–10, suggesting that *d* may vary among individuals.

## 1. Introduction

Noroviruses (NoVs) constitute the genus *Norovirus* in the family *Caliciviridae* [1]. The NoV virion is non-enveloped and icosahedral with a size of 38 nm in diameter [2]. The NoV genome is a linear, non-segmented, single-stranded RNA of positive polarity with a length of 7.5–7.7 kb [3,4]. ORF1, ORF2, and ORF3 are located in this order from the 5′-end to the 3′-end of the genome. ORF1 encodes a polyprotein precursor, which is cleaved into six non-structural proteins (NSs) including RNA-dependent RNA polymerase (RdRp). ORF2 and ORF3 encode major capsid protein (viral protein 1, VP1) and minor capsid protein (VP2), respectively. The NoV capsid is composed of 90 dimers of VP1, which consists of shell (S) and protruding (P) domains forming the core and the spike, respectively [2]. The P domain comprises P1 and P2 subdomains, which form the proximal and distal parts of the spike, respectively. The NoV infection is initiated with binding of P2 subdomain to cellular receptors and/or histo-blood group antigens (HBGAs) [5]. The P2 subdomain is considered to be the major determinant of antigenicity in NoVs [6,7].

Based on the similarity in the amino acid sequence of VP1, NoVs have been classified into 12 genogroups (GI–GX, GNA1, and GNA2) [8]. GI, GII, GIV, GVIII, and GIX NoVs infect humans and constitute human NoVs (HuNoVs) [9]. GI, GII, and GIV have been further classified into genotypes GI.1–GI.9, GII.1–GII.27, and GIV.1–GIV.2, respectively [10]. In the NoV genome, recombination hotspot exists at the junction of ORF1 and ORF2 [11,12]. Thus, genogroups and genotypes represent the classification system for the downstream region of the recombination hotspot. The classification system for the upstream region has also been established based on the similarity in nucleotide sequence encoding RdRp; NoVs have been classified into 10 P-groups (GI.P–GVII.P, GX.P, GNA1.P, and GNA2.P) [8]. GI.P and GII.P have been further classified into P-types GI.P1–GI.P14 and GII.P1–GII.P41, respectively [10].

HuNoVs are responsible for 18% (685 million cases) of acute gastroenteritis (AGE) and 212 thousand deaths worldwide annually [13,14]. Prevalence of HuNoVs in AGE is greater in developed countries (20%) and low-mortality developing countries (19%) than in high-mortality developing countries (14%), suggesting that improvements in sanitation and hygiene may not be sufficient for control and prevention of HuNoVs [15]. It is therefore necessary to develop vaccines as well as drugs against HuNoVs [16,17]. VP1-derived virus-like particles (VLPs), P domain-derived P particles, and recombinant adenoviruses expressing VP1 have been considered as vaccine candidates [18,19,20].

In Japan, the HuNoV season is defined such that season $t/\left(t+1\right)$ indicates the period between September of year t and August of year $\left(t+1\right)$. Multiple genotypes co-circulate every season changing their proportions [21]. Since the protective effect of heterotypic immunity appears to be limited, if there is any [22], it may be desirable to develop multivalent vaccines against HuNoVs by formulating the genotype composition of seed strains to match that of dominant strains in the target season. This process may be facilitated by predicting genotype proportions in the target season.

Attempts have been made for predicting genotype proportions in HuNoV seasons in Japan taking into account herd immunity to VP1 [23,24]. Although directions of changes in genotype proportions could be predicted with a certain degree of accuracy, it was still difficult to predict exact values of genotype proportions. To improve predictions, it was necessary to understand evolutionary mechanisms of genotype proportions. Reportedly, acquisition of novel P-types through recombination was associated with an increase in genotype proportions [25,26,27]. In addition, proliferation efficiency appeared to be genotype-specific [28]. The purpose of the present study was to predict dominant genotypes in HuNoV seasons in Japan taking into account the acquisition of novel P-types through recombination and genotype-specific proliferation efficiency as well as herd immunity to VP1.

## 2. Materials and Methods

### 2.1. Epidemiological Data

Observed genotype frequencies of HuNoVs in Japan have been monitored by the National Institute of Infectious Diseases (NIID) and deposited in the Infectious Agents Surveillance Report (IASR). As of 26 July 2022, observed genotype frequencies were available for the seasons from 2006/2007 to 2020/2021 (Table S1). Only GI and GII strains have been observed in these seasons. Observed genotype frequencies were converted into observed genotype proportions in each season for GI and GII separately. In the present study, proportions were predicted for the genotypes whose observed proportions have exceeded 12.5% in at least one season; i.e., GI.2, GI.3, GI.4, GI.6, and GI.7 for GI and GII.2, GII.3, GII.4, GII.6, and GII.17 for GII, because other genotypes were unlikely to become dominant and predicting many genotype proportions may enhance stochastic errors. Observed proportions were re-calculated for the above genotypes. The proportion of 0 was replaced with 0.0001, which was smaller than all non-0 values, to avoid the situation that predicted genotype proportions would automatically become 0 (Table S2) [29].

### 2.2. Sequence Data

Nucleotide sequences for HuNoVs provided with the isolation year and month were retrieved from the International Nucleotide Sequence Database (INSD) on 2 August 2022. Genotype and P-type were determined for each sequence using the Norovirus Genotyping Tool (version 2.0) [30]. Among the dually typed sequences, those with GI.2, GI.3, GI.4, GI.6, and GI.7 as well as GII.2, GII.3, GII.4, GII.6, and GII.17 (Table S3) were classified based on the isolation season to identify the seasons in which novel P-types were acquired by individual genotypes (Table S4).

Among the nucleotide sequences for HuNoVs provided with the isolation year and month and the genotypes determined above, those containing the entire coding region of VP1 without pre-termination codons, minor gaps, and ambiguous nucleotides were collected. The sequences with GI.2, GI.3, GI.4, GI.6, and GI.7 as well as GII.2, GII.3, GII.4, GII.6, and GII.17 (Table S5) were classified based on the isolation season to be used in the prediction of genotype proportions as described below (Table S6).

### 2.3. Prediction Models

One of the simplest models for changes in genotype proportions may be to assume that the proportion of genotype g in the target season $t/\left(t+1\right)$ ($p_{g\left(t\right)}$) would be the same as those in the immediately preceding (pre-target) season $\left(t-1\right)/t$ ($p_{g\left(t-1\right)}$). This model is called the null model in the present study.

In the fitness model [31], $p_{g\left(t\right)}$ was predicted from $p_{g\left(t-1\right)}$ using the equation:

(1) $${\hat{p}}_{g\left(t\right)}=p_{g\left(t-1\right)}\times e^{f_{g}}$$

where $f_{g}$ is the fitness of g defined as:

(2) $$f_{g}=f_{0}-c_{g}.$$

Here, $f_{0}$ is the constant to ensure $\underset{g}{\sum }{\hat{p}}_{g\left(t\right)}=1$ and $c_{g}$ the fitness cost of g defined as:

(3) $$c_{g}=r_{g}+a_{g}+h_{g}.$$

The term $r_{g}$ denotes the genotype-specific proliferation efficiency and $a_{g}$ denotes the effect of acquisition of novel P-types computed as the product of coefficient a and the cumulative number of acquisitions of novel P-types by g up to the pre-target season ($n_{g\left(t-1\right)}$). The term $h_{g}$ denotes the effect of herd immunity computed as the sum of homotypic immunity to strain i’s belonging to g isolated in the pre-target season elicited by strain j’s isolated from season 2006/2007 to the pre-target season, that is:

(4) $$h_{g}=\sum_{i}^{}\sum_{j}h\times p_{j\left(t_{j}\right)}\times q_{ij}\times \frac{d-\left(t_{i}-t_{j}\right)}{d}$$

where h is the coefficient of immunity and $t_{i}$ and $t_{j}$ are the isolation seasons of i and j, respectively ($t_{i}\geq t_{j}$). The strength of immunity was assumed to be proportional to the observed proportion of j in $t_{j}$ ($p_{j\left(t_{j}\right)}$), and the identity in amino acid sequence of P2 subdomain between i and j ($q_{ij}$) [32]. The immunity was assumed to decline linearly over the duration of the d seasons as indicated by the last term. Since the reported duration of immunity to HuNoVs ranged from several months to a decade [33,34,35], d was assumed to be 0–10. The last term was set to be 0 if d=0 or $t_{i}-t_{j}>d$.

### 2.4. Data Analysis

The genotype proportions in the target season were predicted to be the same as those in the pre-target season in the null model. In the fitness model, parameters a, h, and $r_{g}$ were optimized using the genetic algorithm [36] through minimizing the sum of squared deviations of predicted genotype proportions from observed genotype proportions over the period between season 2007/2008 and the pre-target season. The optimized parameter values were adopted for predicting genotype proportions in the target season.

In the seasons from 2006/2007 to 2020/2021, the observed proportion for the most prevalent genotype ranged from 0.286 to 0.748 (average: 0.511) in GI and from 0.391 to 0.976 (average: 0.630) in GII (Table S2), which appeared to be relatively small for developing vaccines [37]. However, the sum of the observed proportions for the two most prevalent genotypes ranged from 0.571 to 0.939 (average: 0.776) in GI and from 0.713 to 0.986 (average: 0.861) in GII, which were substantially large. Thus, the two most prevalent genotypes were regarded as the dominant genotypes in each season.

Performances of the null and fitness models for predicting dominant genotypes were evaluated retrospectively by setting the target season to be from 2008/2009 to 2020/2021. The numbers of true positives (tp), true negatives (tn), false positives (fp), and false negatives (fn) were counted, and the sensitivity and the specificity were computed by $\frac{tp}{tp+fn}$ and $\frac{tn}{tn+fp}$, respectively, with Matthew’s correlation coefficient (MCC) by $\frac{tp\times tn-fp\times fn}{\sqrt{\left(tp+fp\right)\times \left(tp+fn\right)\times \left(tn+fp\right)\times \left(tn+fn\right)}}$. Fisher’s exact test was conducted to examine whether there was a tendency that dominant and non-dominant genotypes were predicted correctly.

## 3. Results

### 3.1. Dominant Genotypes in GI

The null and fitness models were used for predicting genotype proportions and dominant genotypes in GI for the seasons from 2008/2009 to 2020/2021. Since two genotypes were predicted to be dominant for each of the 13 seasons, 26 genotypes were predicted to be dominant in total. Among the 26 dominant genotypes predicted in the null model, 11 and 15 were observed to be dominant (tp) and non-dominant (fp), respectively, in reality (Table 1 and Table S7). In contrast, among the 39 genotypes predicted to be non-dominant, 15 and 24 were observed to be dominant (fn) and non-dominant (tn), respectively, in reality (Table 1 and Table S7). Thus, the sensitivity and the specificity for predicting dominant genotypes were 0.423 and 0.615, respectively, and the MCC was 0.0385. There was no tendency identified that dominant and non-dominant genotypes were predicted correctly (p=0.478 by Fisher’s exact test).

Using the fitness model with d= 0, the numbers of tp, fp, fn, and tn were 12, 14, 14, and 25, respectively (Table 1 and Table S7). The sensitivity and the specificity for predicting dominant genotypes were 0.462 and 0.641, respectively, and the MCC was 0.103. There was again no tendency identified that dominant and non-dominant genotypes were predicted correctly (p=0.284 by Fisher’s exact test). The performance of the fitness model with d=1 was the same as that of the null model (Table 1 and Table S7). Similar performances were also observed for the fitness model with d= 2–10 (Table S7).

Notably, dominant genotypes were often predicted correctly using the fitness model even when the null model failed. For example, GI.4 and GI.6, which became dominant in the 2016/2017 season, were correctly predicted with d= 3, 4, and 8 (Table S7). Similarly, GI.2 and GI.7, which became dominant in the 2017/2018 season were correctly predicted with d= 9 (Table S7). Thus, the dominant genotypes were also predicted using the fitness model as the union of those predicted with d= 0–10, where the number of predicted genotypes exceeded 26. The numbers of tp, fp, fn, and tn were 22, 21, 4, and 18, respectively (Table 1 and Table S7). The sensitivity and the specificity for predicting dominant genotypes were 0.846 and 0.462, respectively, and the MCC was 0.319. There was a tendency identified that dominant and non-dominant genotypes were predicted correctly ($p=9.35\times 10^{-3}$ by Fisher’s exact test).

### 3.2. Dominant Genotypes in GII

The null model performed better for predicting dominant genotypes in GII than in GI. Over the seasons from 2008/2009 to 2020/2021, the numbers of tp, fp, fn, and tn were 18, 8, 8, and 31, respectively (Table 2 and Table S8). Thus, the sensitivity and the specificity for predicting dominant genotypes were 0.692 and 0.795, respectively, and the MCC was 0.487. There was a tendency identified that dominant and non-dominant genotypes were predicted correctly ($p=1.07\times 10^{-4}$ by Fisher’s exact test).

Using the fitness model with d= 0, the numbers of tp, fp, fn, and tn were 19, 7, 7, and 32, respectively (Table 2 and Table S8). The sensitivity and the specificity for predicting dominant genotypes were 0.731 and 0.821, respectively, and the MCC was 0.551. There was again a tendency identified that dominant and non-dominant genotypes were predicted correctly ($p=1.09\times 10^{-5}$ by Fisher’s exact test). The performance of the fitness model with d=1 was the same as that of the null model (Table 2 and Table S8). Similar performances were also observed for the fitness model with d= 2–10 (Table S8).

As was the case for GI, dominant genotypes were often predicted correctly using the fitness model even when the null model failed. For example, GII.3, GII.17, and GII.2, which became dominant in 2014/2015, 2015/2016, and 2016/2017, respectively, were correctly predicted with some d values (Table S8). When dominant genotypes were predicted using the fitness model as the union of those predicted with d= 0–10, the numbers of tp, fp, fn, and tn were 21, 15, 5, and 24, respectively (Table 2 and Table S8). The sensitivity and the specificity for predicting dominant genotypes were 0.808 and 0.615, respectively, and the MCC was 0.417. There was again a tendency identified that dominant and non-dominant genotypes were predicted correctly ($p=7.58\times 10^{-4}$ by Fisher’s exact test).

## 4. Discussion

### 4.1. Dominant Genotypes in HuNoV Seasons in Japan

In the present study, performances of the null and fitness models for predicting dominant genotypes in HuNoV seasons were evaluated using observed genotype frequencies in Japan and genomic sequences for GI and GII strains. Over the seasons in which observed genotype frequencies were available (from 2006/2007 to 2020/2021), the observed proportion for the most prevalent genotype appeared to be relatively small (0.511 for GI and 0.630 for GII on average) [37], but the sum of the observed proportions for the two most prevalent genotypes was substantially large (0.776 for GI and 0.861 for GII on average). As for vaccines against HuNoVs, mono-valent (GI.1, GII.4), bi-valent (GI.1 and GII.4, GI.3 and GII.4), and tetra-valent (GI.1, GII.3, GII.4, and GII.17) candidates have been under clinical investigations [18,19,20]. However, the above observations suggested that tetravalent vaccines containing the two most prevalent genotypes each of GI and GII may also be a candidate, although these genotypes may need to be predicted.

### 4.2. Predicting Dominant Genotypes in HuNoV Seasons in Japan

The null model performed better for predicting dominant genotypes in GII than in GI; there was a tendency identified that dominant and non-dominant genotypes were predicted correctly in GII but not in GI. This was apparently because observed dominant genotypes were relatively stable in GII; e.g., GII.4 was always included in the observed dominant genotypes over the seasons from 2006/2007 to 2020/2021 [38]. Indeed, GII.4 has been characterized by a higher intra-genotypic variation and a faster antigenic evolution than other genotypes of GI and GII, causing six pandemics and five epidemics since the mid-1990s [7,39]. The performance of the fitness model was similar to that of the null model in both GI and GII irrespective of the assumed value of d (0–10). However, the performance of the fitness model improved when dominant genotypes were predicted as the union of those predicted with d= 0–10; there was a tendency identified that dominant and non-dominant genotypes were predicted correctly in both GI and GII. The relatively high performance for the union suggested that d may vary among individuals [33,34,35]. Although genotype proportions and dominant genotypes in HuNoV seasons in Japan were predicted in the present study, the results obtained may also be applicable to those in other regions, because the genotype composition of HuNoVs circulating in Japan and other regions appeared to be similar [27].

### 4.3. Implications for the Development of HuNoV Vaccines

Although the prediction of dominant genotypes using the fitness model as the union of those predicted with d= 0–10 showed a relatively high performance, 43 and 36 genotypes were predicted to be dominant over 13 seasons for GI and GII, respectively; that is, on average 3.3 and 2.8 genotypes were predicted to be dominant in each season for GI and GII, respectively. Thus, hexavalent vaccines, instead of tetravalent vaccines, may be developed according to the prediction [40]. Such hexavalent vaccines may be effective because the sensitivity for predicting dominant genotypes appears to be high. In fact, in the seasons from 2014/2015 to 2020/2021, 13 and 14 out of 14 observed dominant genotypes were included in the predicted dominant genotypes for GI and GII, respectively. However, the specificity should also be increased to reduce the number of predicted dominant genotypes, because excess doses of vaccines may induce adverse events [41].

### 4.4. Future Directions in Predicting Dominant Genotypes

The fitness model should be improved to increase the specificity for predicting dominant genotypes. In the present study, only the homotypic immunity was taken into consideration and its strength was estimated from the identity in the amino acid sequence of P2 subdomain. Although P2 subdomain may be the major determinant of antigenicity, other regions of VP1 may also elicit protective immunity [6,7]. A model for estimating antigenic distances between HuNoV strains based on structural positions and physicochemical properties of different amino acids in the entire region of VP1 should be developed and incorporated into the fitness model [42]. In addition, heterotypic immunity may prevent [39] or enhance infections through the phenomenon known as original antigenic sin or immune imprinting [43,44]. Thus, the relationship between antigenic distance and homotypic or heterotypic immunity should be clarified and incorporated into the fitness model. Since immunological parameters, such as breadth and duration of immunity, may be different between children and adults, the age distribution of infected individuals may also be incorporated into the fitness model [45]. Furthermore, various genotypes of GI and GII HuNoVs apparently have been escaping from herd immunity through antigenic evolution [46,47]. It would therefore be important to predict not only dominant genotypes but also their antigenicity, i.e., amino acid sequences of VP1, by predicting strain proportions of HuNoVs [31]. Emergence of new strains may also be predicted through integrating probabilities of occurrences for mutations causing amino acid substitutions and recombinations with their effects in the fitness model [48].

## Figures and Tables

**Table 1:** Numbers of predicted and observed dominant and non-dominant genotypes in GI for seasons from 2008/2009 to 2020/2021.

			Observed	
Model	Predicted	Dominant	Non-dominant	Total
Null	Dominant	11	15	26
	Non-dominant	15	24	39
	Total	26	39	65
Fitness (*d* = 0)	Dominant	12	14	26
	Non-dominant	14	25	39
	Total	26	39	65
Fitness (*d* = 1)	Dominant	11	15	26
	Non-dominant	15	24	39
	Total	26	39	65
Fitness (*d* = 0–10) ^1^	Dominant	22	21	43
	Non-dominant	4	18	22
	Total	26	39	65

^1^ Dominant genotypes were predicted as the union of those predicted with *d* = 0–10.

**Table 2:** Numbers of predicted and observed dominant and non-dominant genotypes in GII for seasons from 2008/2009 to 2020/2021.

			Observed	
Model	Predicted	Dominant	Non-dominant	Total
Null	Dominant	18	8	26
	Non-dominant	8	31	39
	Total	26	39	65
Fitness (*d* = 0)	Dominant	19	7	26
	Non-dominant	7	32	39
	Total	26	39	65
Fitness (*d* = 1)	Dominant	18	8	26
	Non-dominant	8	31	39
	Total	26	39	65
Fitness (*d* = 0–10) ^1^	Dominant	21	15	36
	Non-dominant	5	24	29
	Total	26	39	65

^1^ Dominant genotypes were predicted as the union of those predicted with *d* = 0–10.

## Data Availability

The data presented in this study are available on request from the corresponding author.

## Supplementary Materials

The following supporting information can be downloaded at: https://www.mdpi.com/article/10.3390/life13081634/s1. Table S1: Observed genotype frequencies of GI and GII HuNoVs deposited in the IASR; Table S2: Observed genotype proportions of GI.2, GI.3, GI.4, GI.6, and GI.7 as well as GII.2, GII.3, GII.4, GII.6, and GII.17; Table S3: Sequence data of GI.2, GI.3, GI.4, GI.6, and GI.7 as well as GII.2, GII.3, GII.4, GII.6, and GII.17 for which P-types were also determined; Table S4: P-types combined with GI.2, GI.3, GI.4, GI.6, and GI.7 as well as GII.2, GII.3, GII.4, GII.6, and GII.17; Table S5: Sequence data of GI.2, GI.3, GI.4, GI.6, and GI.7 as well as GII.2, GII.3, GII.4, GII.6, and GII.17 containing the entire coding region of VP1; Table S6: Numbers of sequences for GI.2, GI.3, GI.4, GI.6, and GI.7 as well as GII.2, GII.3, GII.4, GII.6, and GII.17 containing the entire coding region of VP1; Table S7: Genotype proportions and dominant genotypes in GI; and Table S8: Genotype proportions and dominant genotypes in GII.
