# Supplementary material for: Predicting Dominant Genotypes in Norovirus Seasons in Japan
Source: Life (Basel). 2023 Jul 27;13(8):1634. doi: 10.3390/life13081634 (PMC10455559; doi:10.3390/life13081634)
Supplement: Supplementary file 1 [file life-13-01634-s001.zip › TableS7.pdf]

Table S7: Genotype proportions and dominant genotypes in GI.

Season	Geno-type	Obs.[a]	Null[b]	*d*[c]										
				0	1	2	3	4	5	6	7	8	9	10
2008/2009	GI.2	0	0	0	5E-06	N.A.[e]	N.A.	N.A.	N.A.	N.A.	N.A.	N.A.	N.A.	N.A.
	GI.3	**0.1961**[d]	0.0625	0.0625	**0.4762**	N.A.	N.A.	N.A.	N.A.	N.A.	N.A.	N.A.	N.A.	N.A.
	GI.4	**0.6078**	**0.7188**	**0.7188**	0.0005	N.A.	N.A.	N.A.	N.A.	N.A.	N.A.	N.A.	N.A.	N.A.
	GI.6	0.1373	**0.2083**	**0.2083**	**0.4882**	N.A.	N.A.	N.A.	N.A.	N.A.	N.A.	N.A.	N.A.	N.A.
	GI.7	0.0588	0.0104	0.0104	0.0351	N.A.	N.A.	N.A.	N.A.	N.A.	N.A.	N.A.	N.A.	N.A.
2009/2010	GI.2	0.0111	0	0	5E-06	1E-05	N.A.	N.A.	N.A.	N.A.	N.A.	N.A.	N.A.	N.A.
	GI.3	0	**0.1961**	**0.1961**	**0.1433**	**0.0717**	N.A.	N.A.	N.A.	N.A.	N.A.	N.A.	N.A.	N.A.
	GI.4	**0.4333**	**0.6078**	**0.6078**	**0.7522**	**0.8586**	N.A.	N.A.	N.A.	N.A.	N.A.	N.A.	N.A.	N.A.
	GI.6	**0.4222**	0.1373	0.1373	0.0685	0.0503	N.A.	N.A.	N.A.	N.A.	N.A.	N.A.	N.A.	N.A.
	GI.7	0.1333	0.0588	0.0588	0.036	0.0194	N.A.	N.A.	N.A.	N.A.	N.A.	N.A.	N.A.	N.A.
2010/2011	GI.2	**0.35**	0.0111	0.0111	0	**0.727**	**0.3939**	N.A.	N.A.	N.A.	N.A.	N.A.	N.A.	N.A.
	GI.3	0.2	0	0	7E-05	8E-06	0	N.A.	N.A.	N.A.	N.A.	N.A.	N.A.	N.A.
	GI.4	0	**0.4333**	**0.4333**	**0.5407**	0.0717	0.1411	N.A.	N.A.	N.A.	N.A.	N.A.	N.A.	N.A.
	GI.6	**0.25**	**0.4222**	**0.4222**	**0.2604**	**0.1333**	**0.3725**	N.A.	N.A.	N.A.	N.A.	N.A.	N.A.	N.A.
	GI.7	0.2	0.1333	0.1333	0.1988	0.068	0.0925	N.A.	N.A.	N.A.	N.A.	N.A.	N.A.	N.A.
2011/2012	GI.2	0.0763	**0.35**	**0.35**	**0.4126**	**0.9628**	**0.9632**	0	N.A.	N.A.	N.A.	N.A.	N.A.	N.A.
	GI.3	**0.2458**	0.2	0.2	0.0528	0.0028	0	**0.9209**	N.A.	N.A.	N.A.	N.A.	N.A.	N.A.
	GI.4	**0.4322**	0	0	8E-05	3E-06	2E-06	0	N.A.	N.A.	N.A.	N.A.	N.A.	N.A.
	GI.6	0.2119	**0.25**	**0.25**	0.1915	0.0127	0.0184	**0.0773**	N.A.	N.A.	N.A.	N.A.	N.A.	N.A.
	GI.7	0.0339	0.2	0.2	**0.343**	**0.0217**	**0.0185**	0.0018	N.A.	N.A.	N.A.	N.A.	N.A.	N.A.
2012/2013	GI.2	0.0144	0.0763	8E-06	0.0611	2E-05	0	0	0	N.A.	N.A.	N.A.	N.A.	N.A.
	GI.3	0.0432	**0.2458**	0.0001	0.2008	7E-05	3E-05	4E-06	0	N.A.	N.A.	N.A.	N.A.	N.A.
	GI.4	**0.1799**	**0.4322**	**0.9239**	**0.4081**	**8E-05**	**0.0528**	**0.9616**	0	N.A.	N.A.	N.A.	N.A.	N.A.
	GI.6	**0.7482**	0.2119	**0.076**	**0.2833**	**0.9998**	**0.9472**	**0.0384**	**1**	N.A.	N.A.	N.A.	N.A.	N.A.
	GI.7	0.0144	0.0339	3E-06	0.0467	2E-05	2E-06	0	0	N.A.	N.A.	N.A.	N.A.	N.A.
2013/2014	GI.2	**0.2373**	0.0144	0	0.0023	0.0018	0	0	0	0.0043	N.A.	N.A.	N.A.	N.A.
	GI.3	0.1186	0.0432	**0.0764**	**0.0485**	**0.0385**	**0.0422**	**0.0311**	**0.0196**	**0.3313**	N.A.	N.A.	N.A.	N.A.
	GI.4	**0.4068**	**0.1799**	0.0287	0.0349	0.0219	**0.9577**	**0.9653**	**0.9754**	**0.6577**	N.A.	N.A.	N.A.	N.A.
	GI.6	0.1186	**0.7482**	**0.895**	**0.9108**	**0.9338**	7E-06	0.0035	0.005	0.0046	N.A.	N.A.	N.A.	N.A.
	GI.7	0.1186	0.0144	0	0.0036	0.0041	2E-05	0	0	0.002	N.A.	N.A.	N.A.	N.A.
2014/2015	GI.2	**0.2883**	**0.2373**	1E-06	**0.3147**	**0.2281**	0.0017	0.0167	5E-06	**0.5006**	**0.1072**	N.A.	N.A.	N.A.
	GI.3	**0.6503**	0.1186	**0.741**	0.1409	**0.2386**	**0.2996**	**0.3202**	**0.3476**	0.0376	0.0104	N.A.	N.A.	N.A.
	GI.4	0.0368	**0.4068**	0.0276	**0.1997**	0.1849	0.1252	0.1859	**0.5203**	**0.226**	0.0706	N.A.	N.A.	N.A.
	GI.6	0.0184	0.1186	0.0476	0.1671	0.1594	0.2058	0.1388	0.0834	0.0149	0.0052	N.A.	N.A.	N.A.
	GI.7	0.0061	0.1186	**0.1837**	0.1777	0.189	**0.3677**	**0.3385**	0.0488	0.221	**0.8066**	N.A.	N.A.	N.A.
2015/2016	GI.2	**0.431**	**0.2883**	0	**0.3145**	9E-06	0.0051	0.0015	**0.6944**	**0.374**	**0.1339**	**0.3036**	N.A.	N.A.
	GI.3	**0.4655**	**0.6503**	**0.9956**	**0.5884**	8E-05	0.2479	0.1133	0.0831	**0.5212**	**0.8569**	0.0518	N.A.	N.A.
	GI.4	0.0603	0.0368	0.0008	0.0392	**0.0993**	**0.251**	**0.3518**	**0.127**	0.0864	0.0084	**0.5844**	N.A.	N.A.
	GI.6	0.0431	0.0184	**0.0021**	0.0448	**0.8993**	**0.487**	**0.4868**	0.089	0.0184	0.0008	0.0593	N.A.	N.A.
	GI.7	0	0.0061	0.0014	0.0132	0.0013	0.0091	0.0466	0.0065	3E-06	0	0.0009	N.A.	N.A.
2016/2017	GI.2	0.1224	**0.431**	1E-06	**0.3414**	**0.2271**	0.0885	0.1296	**0.3713**	**0.465**	**0.4326**	0.1708	**0.3765**	N.A.
	GI.3	0.0612	**0.4655**	**0.9918**	**0.499**	0.1943	0.0624	0.0909	0.0619	0.1492	0.1979	0.0694	0.1907	N.A.
	GI.4	**0.2245**	0.0603	0.0017	0.0601	0.1858	**0.1603**	**0.2255**	0.2209	**0.2735**	**0.3342**	**0.5544**	**0.2502**	N.A.
	GI.6	**0.4082**	0.0431	**0.0065**	0.0993	**0.3926**	**0.6885**	**0.5537**	**0.3458**	0.1123	0.0353	**0.2054**	0.1826	N.A.
	GI.7	0.1837	0	4E-05	0.0002	0.0003	0.0003	0.0003	0.0001	0	0	1E-05	2E-06	N.A.
2017/2018	GI.2	**0.2857**	0.1224	0.082	0.2343	**0.1345**	0.0646	0.0393	**0.1212**	**0.2514**	**0.143**	**0.1156**	**0.5119**	**0.01**
	GI.3	0.2269	0.0612	0.0319	0.1696	0.1315	0.0271	0.0181	0.0114	0.014	0.0121	0.0117	0.009	0.0008
	GI.4	0.1261	**0.2245**	0.0944	0.0428	0.0575	0.0674	0.0548	0.0725	0.0909	0.0988	0.091	0.0003	0.0013
	GI.6	0.0756	**0.4082**	**0.6413**	**0.3108**	**0.5555**	**0.6803**	**0.7777**	**0.7381**	**0.6437**	**0.6745**	**0.7746**	0.002	0.0065
	GI.7	**0.2857**	0.1837	**0.1502**	**0.2425**	0.121	**0.1605**	**0.1101**	0.0569	0	0.0716	0.0071	**0.4767**	**0.9814**
2018/2019	GI.2	**0.6139**	**0.2857**	**0.3626**	0.0543	0.0034	0.1248	0.0523	0.0708	0.0009	**0.5912**	0.0023	**0.3406**	0
	GI.3	0.0198	0.2269	0.262	**0.4838**	**0.7124**	**0.4271**	**0.422**	**0.3751**	**0.4671**	0.1132	**0.4162**	**0.4505**	**1**
	GI.4	0.0693	0.1261	0.0681	0.011	0.0002	0.0105	0.0084	0.0081	9E-05	0.0729	2E-05	0.0028	0
	GI.6	**0.1584**	0.0756	0.009	0.0592	0.0016	0.093	0.0519	0.0612	0.0002	0.0614	0.0006	0.1199	0
	GI.7	0.1386	**0.2857**	**0.2984**	**0.3916**	**0.2823**	**0.3446**	**0.4655**	**0.4848**	**0.5316**	**0.1614**	**0.5809**	0.0863	**2E-05**
2019/2020	GI.2	**0.1667**	**0.6139**	**0.8133**	0.1573	0.0705	**0.1858**	**0.2015**	**0.1157**	**0.1816**	**0.4641**	**0.2377**	0.0298	0.0053
	GI.3	0.0714	0.0198	0.0197	0.081	0.052	0.071	0.0759	0.0806	0.0573	0.0246	0.0293	0.0472	0.0082
	GI.4	**0.6667**	0.0693	0.0345	**0.2107**	**0.1177**	0.0676	0.0686	0.0663	0.0659	0.1446	0.0289	**0.0592**	**0.3378**
	GI.6	0.0714	**0.1584**	0.0175	**0.3421**	**0.6829**	**0.5641**	**0.5553**	**0.6607**	**0.6137**	**0.2992**	**0.6722**	**0.8591**	**0.6436**
	GI.7	0.0238	0.1386	**0.1149**	0.209	0.0768	0.1116	0.0986	0.0767	0.0815	0.0675	0.032	0.0046	0.0052
2020/2021	GI.2	0.12	**0.1667**	**0.2376**	**0.4849**	0.1968	0.1506	0.1849	0.092	0.1599	**0.3179**	0.2923	**0.2811**	0.0342
	GI.3	0	0.0714	0.0886	0.1957	**0.3556**	**0.2753**	**0.4418**	**0.6581**	**0.5476**	0.256	**0.3252**	**0.5599**	**0.2421**
	GI.4	**0.4**	**0.6667**	**0.6187**	0.0043	0.0024	0.0057	0.0147	0.001	0.0066	0.0505	0.0063	0.0004	3E-05
	GI.6	**0.44**	0.0714	0.0237	**0.2514**	**0.3682**	**0.5247**	**0.3283**	**0.2292**	**0.2575**	**0.3503**	**0.3625**	0.152	**0.7227**
	GI.7	0.04	0.0238	0.0313	0.0636	0.0769	0.0437	0.0303	0.0196	0.0284	0.0253	0.0138	0.0066	0.001
2021/2022	GI.2	N.A.	0.12	0.1328	**0.3736**	**0.4889**	**0.2414**	**0.1883**	**0.2377**	**0.3014**	**0.2818**	**0.2542**	**0.3551**	**0.4354**
	GI.3	N.A.	0	8E-05	0.0008	0.0005	0.0005	0.0005	0.0009	0.0014	0.0006	0.0008	0.0013	0.0012
	GI.4	N.A.	**0.4**	**0.2843**	0.0089	6E-05	0.0005	0.0008	0.0002	0.0001	0.001	5E-05	1E-05	2E-06
	GI.6	N.A.	**0.44**	**0.5368**	**0.4516**	**0.4097**	**0.6663**	**0.7568**	**0.7109**	**0.6259**	**0.6823**	**0.7245**	**0.6216**	**0.5507**
	GI.7	N.A.	0.04	0.046	0.1652	0.1008	0.0912	0.0536	0.0503	0.0713	0.0343	0.0204	0.022	0.0127

[a]Observed genotype proportions.

[b]Predicted genotype proportions using the null model.

[c]Predicted genotype proportions using the fitness model with d = 0-10.

[d]Dominant genotypes are bold-faced.

[e]Not applicable.
