# Supplementary material for: Predicting Dominant Genotypes in Norovirus Seasons in Japan
Source: Life (Basel). 2023 Jul 27;13(8):1634. doi: 10.3390/life13081634 (PMC10455559; doi:10.3390/life13081634)
Supplement: Supplementary file 1 [file life-13-01634-s001.zip › TableS8.pdf]

Table S8: Genotype proportions and dominant genotypes in GII.

Season	Geno-type	Obs.[a]	Null[b]	d[c] 0	1	2	3	4	5	6	7	8	9	10
2008	GII.2	0.0519	0.0433	0.1124	0.1272	N.A.[e]	N.A.	N.A.	N.A.	N.A.	N.A.	N.A.	N.A.	N.A.
/2009	GII.3	0.064	**0.1198**	**0.7032**	**0.4007**	N.A.	N.A.	N.A.	N.A.	N.A.	N.A.	N.A.	N.A.	N.A.
	GII.4	**0.6384**[d]	**0.8326**	**0.1839**	**0.4712**	N.A.	N.A.	N.A.	N.A.	N.A.	N.A.	N.A.	N.A.	N.A.
	GII.6	**0.2439**	0.0043	0.0005	0.0008	N.A.	N.A.	N.A.	N.A.	N.A.	N.A.	N.A.	N.A.	N.A.
	GII.17	0.0017	0	3E-05	4E-05	N.A.	N.A.	N.A.	N.A.	N.A.	N.A.	N.A.	N.A.	N.A.
2009	GII.2	**0.318**	0.0519	0.0284	0.031	0.0071	N.A.	N.A.	N.A.	N.A.	N.A.	N.A.	N.A.	N.A.
/2010	GII.3	0.0618	0.064	0.0154	0.0179	0.0073	N.A.	N.A.	N.A.	N.A.	N.A.	N.A.	N.A.	N.A.
	GII.4	**0.6028**	**0.6384**	**0.1267**	**0.1307**	**0.1281**	N.A.	N.A.	N.A.	N.A.	N.A.	N.A.	N.A.	N.A.
	GII.6	0.0175	**0.2439**	**0.7143**	**0.8152**	**0.8539**	N.A.	N.A.	N.A.	N.A.	N.A.	N.A.	N.A.	N.A.
	GII.17	0	0.0017	0.1152	0.0052	0.0037	N.A.	N.A.	N.A.	N.A.	N.A.	N.A.	N.A.	N.A.
2010	GII.2	0.1451	**0.318**	**0.715**	**0.7126**	**0.7121**	**0.6613**	N.A.	N.A.	N.A.	N.A.	N.A.	N.A.	N.A.
/2011	GII.3	**0.467**	0.0618	0.0348	0.036	0.0342	0.0454	N.A.	N.A.	N.A.	N.A.	N.A.	N.A.	N.A.
	GII.4	**0.3843**	**0.6028**	**0.2493**	**0.2505**	**0.2531**	**0.2927**	N.A.	N.A.	N.A.	N.A.	N.A.	N.A.	N.A.
	GII.6	0.0035	0.0175	0.0007	0.0007	0.0006	0.0006	N.A.	N.A.	N.A.	N.A.	N.A.	N.A.	N.A.
	GII.17	0	0	0.0002	0.0002	6E-05	2E-06	N.A.	N.A.	N.A.	N.A.	N.A.	N.A.	N.A.
2011	GII.2	**0.1344**	0.1451	0.0092	0.0375	0.0118	0.0033	0.0002	N.A.	N.A.	N.A.	N.A.	N.A.	N.A.
/2012	GII.3	0.0438	**0.467**	**0.9752**	**0.9047**	**0.952**	**0.9788**	**0.9989**	N.A.	N.A.	N.A.	N.A.	N.A.	N.A.
	GII.4	**0.781**	**0.3843**	**0.0155**	**0.0577**	**0.0362**	**0.0179**	**0.0009**	N.A.	N.A.	N.A.	N.A.	N.A.	N.A.
	GII.6	0.0408	0.0035	3E-05	8E-05	4E-05	5E-06	2E-06	N.A.	N.A.	N.A.	N.A.	N.A.	N.A.
	GII.17	0	0	0.0001	5E-05	3E-05	0	0	N.A.	N.A.	N.A.	N.A.	N.A.	N.A.
2012	GII.2	**0.0521**	**0.1344**	**0.1449**	0.0116	8E-05	0.0003	**0.1205**	0.0647	N.A.	N.A.	N.A.	N.A.	N.A.
/2013	GII.3	0.0165	0.0438	0.0065	**0.9433**	**0.8338**	**0.572**	0.0364	0.0023	N.A.	N.A.	N.A.	N.A.	N.A.
	GII.4	**0.9074**	**0.781**	**0.8225**	**0.0436**	**0.1603**	**0.427**	**0.8286**	**0.6982**	N.A.	N.A.	N.A.	N.A.	N.A.
	GII.6	0.0223	0.0408	0.0199	0.0012	0.0057	0.0008	0.0091	**0.2348**	N.A.	N.A.	N.A.	N.A.	N.A.
	GII.17	0.0017	0	0.0061	0.0003	0.0002	0	0.0053	5E-05	N.A.	N.A.	N.A.	N.A.	N.A.
2013	GII.2	0.0266	**0.0521**	0.0448	**0.1252**	**0.1763**	0.0266	**0.3486**	0.0233	0.0195	N.A.	N.A.	N.A.	N.A.
/2014	GII.3	0.0577	0.0165	0.0021	0.0469	0.031	**0.8878**	0.0937	0.001	0.006	N.A.	N.A.	N.A.	N.A.
	GII.4	**0.587**	**0.9074**	**0.8524**	**0.7177**	**0.7343**	**0.0855**	**0.4401**	**0.901**	**0.9374**	N.A.	N.A.	N.A.	N.A.
	GII.6	**0.3269**	0.0223	0.0096	0.0197	0.011	0.0001	3E-05	0.0112	0.0115	N.A.	N.A.	N.A.	N.A.
	GII.17	0.0018	0.0017	**0.0911**	0.0906	0.0474	2E-06	0.1175	**0.0636**	**0.0256**	N.A.	N.A.	N.A.	N.A.
2014	GII.2	0.0029	0.0266	0.0259	0.0337	0.0748	0.1297	0.0604	**0.2635**	0.0249	0.0053	N.A.	N.A.	N.A.
/2015	GII.3	**0.2711**	0.0577	0.0084	0.0978	0.0813	**0.4708**	0.2857	0.157	**0.134**	0.0649	N.A.	N.A.	N.A.
	GII.4	**0.5067**	**0.587**	**0.6117**	**0.6863**	**0.6554**	**0.364**	**0.3528**	**0.3298**	**0.7104**	**0.1776**	N.A.	N.A.	N.A.
	GII.6	0.0086	**0.3269**	**0.1898**	**0.1412**	**0.1263**	0.0263	**0.3012**	0.2497	0.124	**0.7516**	N.A.	N.A.	N.A.
	GII.17	0.2107	0.0018	0.1643	0.0411	0.0622	0.0093	0	1E-06	0.0066	0.0006	N.A.	N.A.	N.A.
2015	GII.2	0.0488	0.0029	0.0002	0.0009	0.0021	0.0027	0.0018	1E-05	0.0011	0	3E-05	N.A.	N.A.
/2016	GII.3	0.1868	**0.2711**	0.0008	0.0518	0.0641	0.0814	**0.1259**	0.0023	0.0124	0.0153	0.0505	N.A.	N.A.
	GII.4	**0.5294**	**0.5067**	**0.0262**	**0.1654**	**0.2757**	**0.1988**	**0.7518**	**0.976**	**0.8341**	**0.7666**	**0.0515**	N.A.	N.A.
	GII.6	0.0266	0.0086	0.0001	0.0015	0.0005	0.0002	0.0016	4E-05	0.0003	0.0003	1E-05	N.A.	N.A.
	GII.17	**0.2084**	0.2107	**0.9727**	**0.7805**	**0.6577**	**0.7169**	0.1189	**0.0216**	**0.1521**	**0.2178**	**0.8979**	N.A.	N.A.
2016	GII.2	**0.675**	0.0488	0.0594	0.0436	0.0505	0.0968	0.0314	**0.1464**	**0.1389**	0.0245	0.0559	**0.0026**	N.A.
/2017	GII.3	0.023	0.1868	0.0737	0.1609	0.0661	0.0867	0.0486	0.1251	0.1034	0.1221	0.0598	0.0004	N.A.
	GII.4	**0.1933**	**0.5294**	**0.5981**	**0.5241**	**0.7431**	**0.7003**	**0.8475**	**0.6278**	**0.6381**	**0.6949**	**0.7997**	**0.9962**	N.A.
	GII.6	0.0568	0.0266	0.0168	0.0179	0.0049	0.002	0.0105	0.0147	0.0149	0.0187	0.0005	0.0003	N.A.
	GII.17	0.0519	**0.2084**	**0.2521**	**0.2535**	**0.1354**	**0.1143**	**0.062**	0.086	0.1047	**0.1398**	**0.0841**	0.0006	N.A.
2017	GII.2	**0.3403**	**0.675**	**0.7738**	**0.1217**	**0.2686**	**0.2691**	**0.0357**	**0.0056**	**0.0176**	**0.0316**	**0.0991**	**0.8891**	**0.9433**
/2018	GII.3	0.0335	0.023	0.0083	0.04	0.0245	0.0253	0.009	0.0029	0.007	0.0051	0.0089	0.0026	0.0003
	GII.4	**0.5124**	**0.1933**	**0.1489**	**0.7054**	**0.6101**	**0.6414**	**0.9309**	**0.9859**	**0.9671**	**0.9541**	**0.8817**	**0.0691**	**0.0425**
	GII.6	0.0161	0.0568	0.0239	0.0292	0.0144	0.0056	0.0138	0.0032	0.0049	0.0045	0.0015	0.0345	0.0125
	GII.17	0.0978	0.0519	0.0451	0.1036	0.0823	0.0586	0.0106	0.0024	0.0034	0.0048	0.0088	0.0048	0.0013
2018	GII.2	**0.2837**	**0.3403**	**0.166**	**0.2815**	**0.1252**	**0.0989**	**0.0357**	**0.0273**	**0.0195**	**0.0332**	**0.0468**	**0.0457**	**0.0384**
/2019	GII.3	0.1565	0.0335	9E-05	0.0491	0.0309	0.0354	0.0238	0.0097	0.0156	0.0234	0.0187	0.0076	0.005
	GII.4	**0.4291**	**0.5124**	**0.8148**	**0.5105**	**0.7158**	**0.7661**	**0.9017**	**0.9462**	**0.9504**	**0.926**	**0.9221**	**0.9282**	**0.9367**
	GII.6	0.0699	0.0161	0.0002	0.0116	0.0037	0.0022	0.0049	0.0022	0.0022	0.003	0.0007	0.013	0.0107
	GII.17	0.0608	0.0978	0.0189	0.1472	0.1243	0.0973	0.0339	0.0146	0.0123	0.0144	0.0118	0.0054	0.0092
2019	GII.2	**0.3537**	**0.2837**	**0.2534**	**0.2561**	**0.255**	0.1184	0.0529	0.0295	0.0167	0.0243	0.0352	0.0426	**0.1934**
/2020	GII.3	0.1434	0.1565	0.0825	0.1489	0.1311	**0.1473**	**0.1235**	**0.0437**	**0.0584**	**0.0746**	**0.08**	0.0366	0.0176
	GII.4	**0.3913**	**0.4291**	**0.514**	**0.471**	**0.4946**	**0.6409**	**0.7595**	**0.8946**	**0.9057**	**0.88**	**0.874**	**0.8089**	**0.7783**
	GII.6	0.0494	0.0699	0.0544	0.0409	0.0249	0.009	0.0195	0.0152	0.009	0.011	0.0031	**0.1076**	0.0098
	GII.17	0.0623	0.0608	0.0957	0.0831	0.0943	0.0843	0.0446	0.017	0.0103	0.0101	0.0076	0.0043	0.0009
2020	GII.2	**0.6191**	**0.3537**	**0.3487**	**0.2855**	**0.2773**	**0.2332**	0.1142	**0.0688**	0.0791	0.0839	**0.1361**	**0.1584**	**0.2741**
/2021	GII.3	0.0217	0.1434	0.0822	0.1433	0.1127	0.1202	**0.1181**	0.0616	**0.1159**	**0.1025**	0.0924	0.04	0.1943
	GII.4	**0.222**	**0.3913**	**0.4424**	**0.4611**	**0.4981**	**0.5473**	**0.6825**	**0.8225**	**0.7584**	**0.7815**	**0.7475**	**0.6748**	**0.5215**
	GII.6	0.0199	0.0494	0.0355	0.0263	0.0168	0.008	0.0173	0.0096	0.0129	0.0091	0.0056	0.1197	0.0092
	GII.17	0.1173	0.0623	0.0912	0.0839	0.0951	0.0913	0.068	0.0375	0.0337	0.023	0.0184	0.007	0.0009
2021	GII.2	N.A.	**0.6191**	**0.6412**	**0.3673**	**0.3767**	**0.3202**	**0.4556**	**0.5453**	**0.5236**	**0.5765**	**0.5503**	**0.7064**	**0.4588**
/2022	GII.3	N.A.	0.0217	0.0092	0.0312	0.0219	0.0226	0.0156	0.0085	0.0227	0.0251	0.0241	0.0194	**0.3817**
	GII.4	N.A.	**0.222**	**0.1991**	**0.4309**	**0.4235**	**0.4943**	**0.3821**	**0.3019**	**0.3332**	**0.3591**	**0.3886**	**0.2644**	0.153
	GII.6	N.A.	0.0199	0.0111	0.0132	0.0064	0.0031	0.0137	0.0121	0.0088	0.0022	0.0011	0.007	0.0049
	GII.17	N.A.	0.1173	0.1394	0.1574	0.1715	0.1598	0.1331	0.1322	0.1118	0.0371	0.0358	0.0028	0.0016

[a]Observed genotype proportions.

[b]Predicted genotype proportions using the null model.

[c]Predicted genotype proportions using the fitness model with $d = 0\text{-}10$.

[d]Dominant genotypes are bold-faced.

[e]Not applicable.
